# Cholera Microbes, Hahnemann, and Dr. Dudgeon

**Published:** 1885-01

**Authors:** P. P. Wells

**Affiliations:** Brooklyn


### HOMŒOPATHIC PHYSICIAN.

A MONTHLY JOURNAL OF MEDICAL SCIENCE.

"If our school ever gives up the strict inductive method of Hahnemann, we are lost, and deserve only to be mentioned as a caricature in the history of medicine."—CONSTANTINE HERING.

Vol. V. JANUARY, 1885. No. 1.

### CHOLERA MICROBES, HAHNEMANN, AND DR. DUDGEON.

"It is the easiest thing in the world to be mistaken."—*Old Proverb.*

On page 272 of Vol. IV. of HOMŒOPATHIC PHYSICIAN, I have said, speaking of the cure and prophylaxis of cholera as discovered and published by Hahnemann, "*and yet, be it remembered, with no thought of 'microbes' he discovered the cure.*" Commenting on this, Dr. Dudgeon says, "*But surely, for once he has made a mistake.*" No doubt we have made many, but we do not admit the statement that the discovery of the curatives and prophylaxis of cholera were made "with no thought of microbes" is to be rightfully added to the number. If it be, then we have only to thank Dr. D. for so kindly calling our attention to it, and to assure him that his kindness shall be no offense to us, but rather "an excellent oil to our head." Let us see, if we may, who "has made a mistake."

The idea that Hahnemann was led to these discoveries by the logical teaching of his already discovered universal law of healing has been so long in my mind as a part of my treasured traditional history that I have now no recollection of its origin—of how it came into my mind. It may have been told me by my late excellent friend Haynel, who, more than any man, would be likely to know the facts in the case. He told me many things, but I do not remember, positively, this as one of them. So if the statment be an error I cannot charge it on him. It may be I gathered the fact from the *Archiv fur Homœopatische Heilkunst*, Band. XI, Th. 1, p. 122 *et seq.*, from a paper entitled "*Heilung der Asiatische Cholera und Schutzuny vor desselben*,* by Samuel Hahnemann. This volume has been on my library shelf more than forty years, has been in my hands many times in these years, and it is now before me. It was in this article that the illustrious master first gave to the world his discovered cure and protection of and from this fearful pestilence. I am the more inclined to refer my long carried belief of the fact which Dr. D. thinks "a mistake" to this source, as while the volume now lies open before me, I fail to find in it any, even the slightest, allusion to "microbes" as a suggestion of either cure or protection. Indeed, there is no mention or allusion to "microbes" in the paper, not even the faintest. Could this have been the case if an idea of these had in any way aided the writer in his discovered cure and protection? Would it have been like him to have so utterly ignored so important a fact as that which had been the index pointing to his so invaluable conclusion? Not so. And this is confirmed to us by this paper, which starts with the fact which seems to have first called the attention of the master to camphor as a curative of the disease, and this fact was not a "microbe," but a recipe, that in Dünaburg was found so successful against Asiatic cholera that of ten sick treated by it only one died, and that the principal ingredient in this recipe was camphor. He says the success with camphor would have been greater if it had been given without mixture with other drugs, which no doubt is true.

> "But," says Dr. D., "not only was Hahnemann a believer in an organized germ being the cause of cholera, his recommendation of camphor was partly, at least, founded on his belief that it was the true germicide of the cholera microbe."

We have said nothing of Hahnemann's *belief* one way or the other, and therefore have "made no mistake" in the matter, and therefore are not called on to correct the mistake of another, if he has made one. At the time he published his cure and prophylaxis he does not seem to have had any belief in these organized germs, at least he says nothing of such belief. He would hardly have been silent on so important a matter, and the more if it had been an efficient factor in leading to the discovery of protection from attacks of cholera. Whatever may have been his belief in "organized germs" when he wrote his cure and protection, it is certain he did not then regard camphor as

---

* Cure of Asiatic cholera and its prophylaxis.

"the true germicide of the cholera microbe." He has left us in no uncertainty as to his view of camphor as a prophylactic. On page 127 of *Archiv* [loc. cit.] he says expressly, "Camphor cannot protect the healthy from attacks of cholera." So it does not then appear that he was even "partly" led to recommend camphor by his confidence in it as a germicide.

How then are we to explain Hahnemann's expressed belief in "organized germs" as connected with the cause of cholera? He says nothing of these in his paper on cure and protection, but in a paper written *subsequently* to this, and published in pamphlet form, *on another subject, viz.:* "The Cause and Prevention of Asiatic Cholera," he no doubt expressed his belief in the germs as stated by Dr. D. He in this pamphlet took part in the discussion, at the time carried on in rather a lively manner, of the contagious or non-contagious nature of the epidemic then prevailing. Hahnemann took the affirmative as to its contagious character, and seems to have regarded his then hypothetical germs as somehow responsible for the production of the disease. Their existence then was only hypothetical, though he says of this it is undoubtedly true that they exist. This certainly is a strong expression to use as to these germs while as yet their existence had not been proved. Their supposed existence seems to have been wholly an idea subsequent to his paper on cure and protection, and to have been used by him to explain and enforce his idea of the contagious nature of the disease and the mode of its propagation. The hypothesis of the existence of the organized germs of Hahnemann's day may now be accepted as a proved fact, and that is all which has been proved. That these *cause* the cholera is another fact not yet proved. If the idea of their causation be not negatived by the failure of all attempts to arrest the present epidemic by acting on these microbes with agents supposed to be destructive of their life, then add the other fact which we have mentioned before, that the epidemic of 1829 crossed the Indian peninsula against the constant blowing of the southwest monsoon, its progress being there and then only and always from northeast to southwest,* and their acceptance as a cause of the disease, in the excretions of which they are found and abundant, becomes impossible.

In conclusion, we think we are justified in saying, if Dr. D. will look at the facts here given and compare them

---

* Copeland's *Cyclopedia of Practical Medicine*, Vol. II, Article "Cholera," p. 318.

with our statement, that microbes had no part in directing Hahnemann's attention to his discovered cure and protection, he will admit, as we claim, that we have made no mistake. But how of his statement, that Hahnemann believed camphor to be "the true germicide," when he says, *Archiv*, p. 127 [loc. cit.], "Camphor kann noch Gesunde vor der Cholera im voraus nicht Schützen, Sondern bloss jenes Kupferpräparat," etc.* Now, in whose eye is the mote? P. P. WELLS.

BROOKLYN, November 18th, 1884.

### THE CHOLERA.

*Still, camphor cannot protect the healthy from cholera, but only some preparation of copper.

